# The Interplay of Economic Resources, Family Decision Power, and Gender Ideology in China

**DOI:** 10.1007/s11205-025-03761-0

**Published:** 2025-12-23

**Authors:** Matthew Henglong Luo, Man-Yee Kan

**Affiliations:** Department of Sociology, University of Oxford, 42-43 Park End Street, Oxford, OX1 1JD UK

**Keywords:** Gender ideology, Economic dependence, Family decision power, Division of labor, China

## Abstract

Gender ideology, the support for a gendered division of paid work and family responsibilities, is a crucial ideological indicator in investigating gender qualities at the couple level. In contrast, economic dependence measures the actual income disparities at the couple level. The two concepts are intertwined with and mutually reinforce each other within marriage. In this study, we propose a mechanism where intra-family decision power interacts with economic dependence to shape gender ideologies and empirically examine it in China. Drawing on data from the China Family Panel Studies (CFPS), our analysis suggests that married women in dual-earner families who adhere to traditional gender ideologies are more likely to be economically dependent on their husbands. On the other side of the loop, a woman’s economic dependence interacts with her intra-family decision power to shape her gender ideology. Intra-family decision power moderates the relationship between economic dependence and gender ideologies so that wives with relatively high economic independence and decision power hold the least traditional gender ideologies, while those with high economic dependence and decision power exhibit the most traditional gender ideologies. These findings are discussed in terms of their implications for gender disparities in the division of labor and the development of gender ideologies.

## Introduction

In modern China, as in many other East Asian and Western societies, women increasingly have equal educational opportunities as men. However, a gendered division of labor persists: women continue to perform more domestic work while men are more engaged in paid work (Kan et al., 2022). Central to this pattern are two important concepts, gender ideology and economic dependence, respectively measuring the internal support/acceptance and economic outcomes of the gendered division of labor. Focusing on married and employed women in dual-earner families, we uncover a pattern in which gender ideology and individual economic resources interact in modern China—a society where, as previous research indicates, traditional gender ideology is still prevalent (e.g., Chen, 2018; Ji et al., 2017; Yeung & Hu, 2016), and the gender disparity in labor participation outcomes has been worsening (Chi & Li, 2014; Gustafsson & Li, 2000; Kan & He, 2018; Kan & Zhou, 2022; Wu & Zhou, 2015).

Prior studies have provided valuable insights into how institutional factors, such as the collapse of the *danwei* system, the marketization of public services, and the labor market environment, shape the gendered division of labor and the acceptance of related ideologies (e.g., He & Wu, 2017, 2021; Ji et al., 2017; Ji & Wu, 2018; Kan et al., 2021; Kan & Zhou, 2022). This study extends this line of literature by incorporating the role of intra-family decision power (i.e., who holds authority over major household domains, e.g., large purchases, children’s schooling, housing decisions, and financial management) and illustrates how economic resources interact with decision power to shape gender ideologies.

Using data from the China Family Panel Studies (CFPS), our analysis suggests that married women who hold traditional gender ideologies are more likely to be economically dependent on their husbands. Meanwhile, we show that economic dependence interacts with intra-family decision power, influencing gender ideology: Wives with relatively high economic *independence* and decision power report the least traditional gender ideologies, while those with high economic *dependence* and decision power report the most traditional ideologies, with other groups falling in between.

Our study makes two major contributions to the literature. First, it contributes to the growing body of literature that utilizes internalized social norms to explain gender inequalities (e.g., Cech, 2013a, b; Khoudja & Fleischmann, 2018). While previous studies have provided crucial insights into how norms and ideologies influence individual career decisions and labor participation in Anglophone and European countries, we extend this line of works by (a) focusing on employed wives, (b) measuring relative income within couples—i.e., economic dependence, and (c) providing empirical evidence from China, an East Asian country.

Second, contributing to the literature on gender ideology (e.g., Bolzendahl & Myers, 2004; Cotter et al., 2011; N. J. Davis & Robinson, 1991; Grunow et al., 2018; Rhodebeck, 1996), we examine how wives’ gender ideology responds to whether economic resources translate into decision-making power within the family. We distinguish *alignment*—when relative resources map onto authority—from two *discrepancies*. We term “penalty” the case of *economic independence without power* (low dependence but low decision-making power); this blocked translation is associated with weaker support for egalitarian ideology. We term “bonus” the case of *economic dependence with power* (high dependence but high decision-making power); this compensatory arrangement is associated with greater acceptance of traditional gender roles. Our results are consistent with both patterns, indicating that distributions of resources and authority shape ideological commitment.

Finally, we offer important practical implications for understanding gender inequalities in modern China. Since the economic reforms of the 1980 s, the country has seen an increasingly gendered division of labor, as illustrated by the gender earnings ratio falling below 70% (Chi & Li, 2014; Gustafsson & Li, 2000), a decline in women’s labor force participation to 62% (Kan & He, 2018; Kan & Zhou, 2022), and women spending 40% less time in the labor sphere but 200% more time in the domestic sphere than men (Kan et al., 2022) in the 21 st century. While many factors contribute to persistent or worsening gender disparities in the labor division, our study highlights the intricate dynamics of economic dependence and family decision-making power in this pattern.

## Theory and Hypotheses

### Supply Side and Gender Inequalities

The supply-side approach to understanding gender disparities in the labor market, while encompassing various contexts and outcomes, begins with the premise that individual choices are central to these disparities. However, the implications of these choices for inequality can vary depending on the underlying assumptions about individuals.

On one hand, the sociological perspective emphasizes the powerful role of socialization and cultural influences in shaping individual choices (Bittman et al., 2003; Ridgeway & Correll, 2004; Ridgeway & Smith-Lovin, 1999). This approach is rooted in the idea that internalized values and external social pressures guide individual decisions, ultimately leading to gender disparities (Cech, 2013b, 2016; Horne & Mollborn, 2020; Seron et al., 2016).

In contrast, the neoclassical economics approach also attributes gender disparities to individual choices but views these choices as utility-maximizing decisions made at either the household level (Becker, 1981) or the individual level (Lundberg & Pollak, 1993, 1996; Manser & Brown, 1980; McElroy & Horney, 1981). From a household perspective, when a woman opts to forego personal income, she is presumed to receive compensatory benefits elsewhere, such as household wealth, satisfaction from fulfilling family responsibilities, or the happiness derived from caregiving work (Folbre, 1995). From an individual perspective, a woman may accept gender disparities in income and in the division of labor because she perceives this arrangement as more beneficial and rational than alternatives like divorce (Chiappori et al., 2017; Grossbard-Shechtman, 1984; Manser & Brown, 1980; McElroy & Horney, 1981; Stevenson, 2007; Stratton, 2020).

Previous studies adopting either the sociological or neoclassical approaches provide rich insights, but they have largely run in parallel, leaving little direct dialogue to explore the potential interaction between internal values and economic benefits. Focusing on gender ideology and economic independence, our study integrates both perspectives. First, building on the relationship between social norms and the traditional division of labor, we hypothesize that traditional gender ideology is associated with married women’s economic dependence. Second, drawing on the concepts of economic rationality and bargaining power within households, we hypothesize an interaction effect between individual relative economic resources and intra-family decision power in shaping gender ideologies.

### Gender Ideology, Division of Labor, and Economic Dependence

Social norms influence individual behavior through two primary mechanisms: internalization and sanctions (Horne & Mollborn, 2020). Simply put, people adhere to norms either because they have internalized the associated values or because they seek to avoid social consequences by conforming to others’ opinions and judgments. In the context of this study, the focus is on the internalized aspect of norms because we aim to investigate gender ideology.

While acknowledging the multidimensional nature of gender ideology (e.g., Begall et al., 2023; Cotter et al., 2011; Grunow et al., 2018; Knight & Brinton, 2017), we align with Davis and Greenstein (2009, p. 87), who define gender ideology as “individuals’ levels of support for a division of paid work and family responsibilities based on the belief in gendered separate spheres.” When traditional gender ideology strongly influences decisions regarding the division of labor within households, married women are likely to participate in the labor market in a way that aligns with the homemaking role. Consequently, at the couple level, the wife often becomes economically dependent on the husband.

In modern China, the relationship between traditional gender ideologies and married women’s economic dependence is particularly pronounced due to the prevalent model of male breadwinning and female homemaking. Since the economic reforms, the labor market has increasingly favored men over women, and traditional divisions of labor have become more common. The gender earnings ratio in urban areas declined from over 85% in the 1980 s to below 70% by the late 2000 s (Chi & Li, 2014; Gustafsson & Li, 2000), while women’s labor force participation rate decreased from 72% in the 1990 s to 62% in 2020 (Kan & He, 2018; Kan & Zhou, 2022; Wu & Zhou, 2015). The collapse of the *danwei* system and the marketization of public services forced many mothers to exit the labor market or accept lower-paying jobs to fulfill homemaking and caregiving responsibilities (He & Wu, 2017; Ji & Wu, 2018; Kan & Zhou, 2022). Furthermore, without effective family policies, men’s competitive advantages have become more pronounced in the post-reform market, while women face more barriers to participating in paid employment and advancing their careers (Ji et al., 2017; Kan et al., 2021; Kan & He, 2018; Zhou et al., 2022). In this context, many Chinese women’s division of labor is driven by traditional gender ideology, leading to economic dependence on their spouses. Therefore, we propose the following hypothesis:

- Hypothesis 1 (H1): Married women’s traditional gender ideology is positively associated with their economic dependence on their spouses.

### Formation of Gender Ideology: Experiences and Benefits

While ideologies can guide individual behaviors, individual behaviors and experiences can, in turn, shape ideologies. Previous studies have identified numerous factors that influence individual gender ideology (e.g., Cotter et al., 2011), which can be broadly categorized into two types: experience-based and interest-based factors (Bolzendahl & Myers, 2004; Grunow et al., 2018).

The experience-based perspective suggests that individuals form their beliefs about gender roles and the division of labor based on their life experiences (Bolzendahl & Myers, 2004). For instance, women who participate in the workforce and pursue higher education often encounter situations that foster feminist and egalitarian attitudes. They are exposed to feminist ideas and interact with people who hold egalitarian beliefs (Davis & Robinson, 1991; Rhodebeck, 1996). These experiences, or the absence of such experiences, contribute to the development of different gender ideologies.

In contrast, the interest-based perspective posits that individuals’ gender ideologies align with their self-identified interests, such as economic gains, prestige, and personal fulfillment. When an egalitarian ideology aligns with personal goals, individuals are more likely to support it, and vice versa (Bolzendahl & Myers, 2004; Davis & Robinson, 1991).

Building on both perspectives, we focus on a particular personal interest and experience: the alignment or discrepancy between relative economic resources and decision-making power within the household. In particular, we treat wives’ gender ideology as responsive to the alignment between their economic dependence (versus independence) and intra-household decision power.

According to the bargaining model and exchange theory, there is an expected association between personal resources and bargaining power within households (Cook & Emerson, 1987; Lundberg & Pollak, 1993). Similarly, resource theory suggests that the power dynamics within couples are based on the relative personal resources of each partner (e.g., Bittman et al., 2003; Brayfield, 1992; Brines, 1994; Gupta, 2007; Kanji & Schober, 2014; Lennon & Rosenfield, 1994). Together, resource and bargaining perspectives predict that relative resources should translate into decision authority—either low dependence with high power (egalitarian alignment) or high dependence with low power (traditional alignment). By contrast, discrepancy arises when this mapping is violated: (a) penalty—low dependence but low power; and (b) bonus—high dependence but high power.

*Penalty (independent but low power).* When economic independence fails to convert into decision power, the blocked translation undermines status claims associated with resources. We expect less egalitarian ideology via two pathways: (a) dissonance reduction (shifting beliefs to fit a non-egalitarian reality) and (b) legitimation of the status quo to make the arrangement feel proper and stable. To elaborate on (a), at the cognitive level, cognitive dissonance predicts that inconsistencies between one’s resources and actual power invite belief adjustment to restore consonance (Festinger, 1962). For (b), system-justification entails that people often legitimize arrangements—even unequal ones—when those arrangements are stable or beneficial on other margins (Jost, 2019; Jost & Banaji, 1994; Zelditch, 2001).

*Bonus (dependent but high power).* At the dyadic level of social exchange, compensated exchange refers to the provision of benefits in one domain (e.g., decision authority, respect, status) that offset costs in another (e.g., economic dependence), thereby sustaining cooperation under unequal conditions (Blau, 1964; Emerson, 1962, 1976; Homans, 1958). When economic dependence coexists with decision power, the arrangement functions as a compensated exchange: authority and respect act as side-payments that make dependence acceptable and even desirable. Endorsing more traditional ideology helps legitimate role complementarity and secures the ongoing exchange.

Moreover, drawing on Bourdieu’s view on class reproduction and symbolic capital (Bourdieu, 1984, 1986), in some middle- and upper-class milieus, economic dependence (e.g., full-time homemaking) can operate as symbolic capital—a visible sign of a family’s position. Decision authority over large expenditures, schooling, or assets then becomes part of the compensatory bundle that sustains the arrangement. Embracing traditional ideology in such contexts can be understood as aligning beliefs with a classed strategy of status reproduction.

**Fig. 1:**
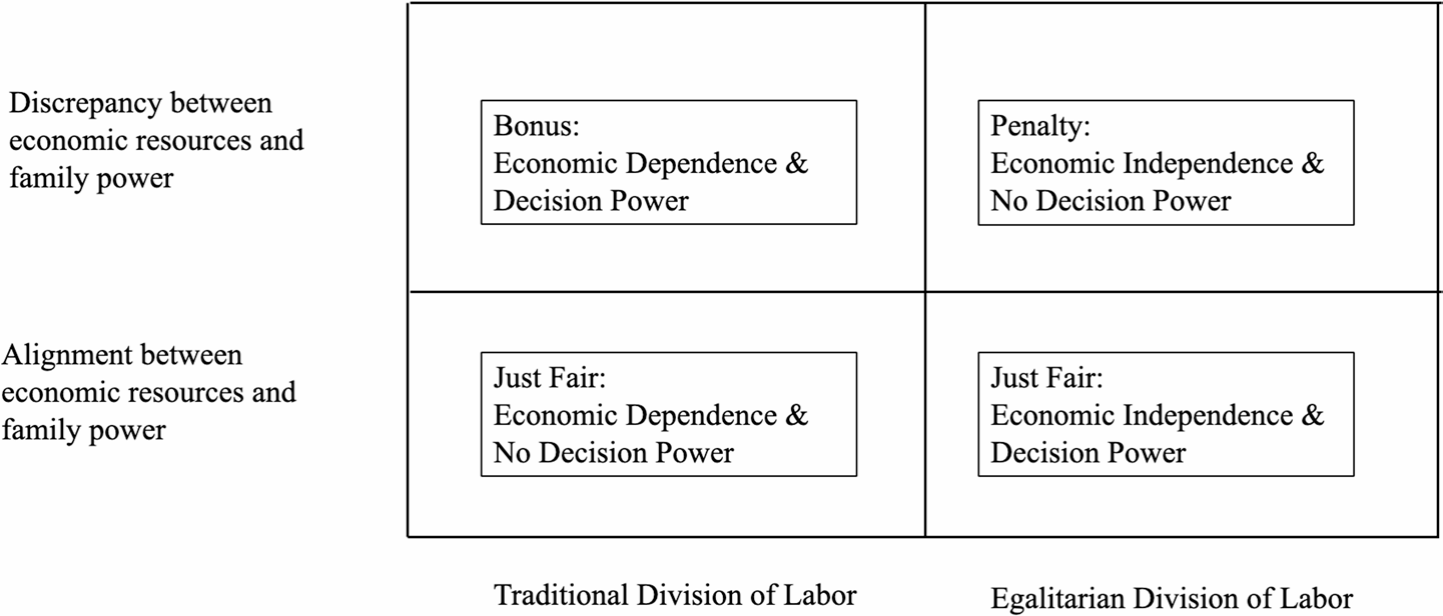
Conceptual Framework of the Discrepancy and Alignment between Economic Dependence and Family Decision Power

To summarize, relative to aligned counterparts, discrepancy is expected to shift ideology in mechanism-consistent directions. Specifically, among economically independent wives (low dependence), lower decision power (greater penalty) is associated with less egalitarian ideology relative to aligned peers (independent + high power). Among economically dependent wives (high dependence), higher decision power (greater bonus) is associated with more traditional ideology relative to aligned peers (dependent + low power). Figure 1 illustrates this alignment/discrepancy conceptual framework.

Is it common for wives to experience discrepancies between relative economic resources and intra-household decision power in modern China? On the one hand, it is not uncommon for economically dependent wives to make important family decisions in China. Previous studies show that although urban wives in China typically contribute less financially to the household than their husbands, their decision-making power often exceeds their corresponding income contribution (e.g., Pimentel, 2006). Zuo and Bian (2005) propose a codependent framework in which couples determine marital power to maintain relational harmony. Shu et al. (2013), drawing on this framework, argue that wives’ disproportionate domestic work, while resulting in economic dependence, is viewed as a contribution to the household and helps them gain decision-making power beyond mundane aspects.

On the other hand, it is not surprising that wives with relatively high economic independence may still lack the final say within the family in a patriarchal society like modern China. While there are few studies specifically focusing on economically independent wives in China, broader literature suggests that although a wife’s relative socioeconomic resources can bring some marital power (e.g., Carlsson et al., 2009; Cheng & Xie, 2023; Qian & Jin, 2018; Xu & Lai, 2002), the predominant pattern remains that husbands hold more decision-making power. Additionally, empirical evidence shows that many dynamics among dual-earner families in China—such as marital quality, happiness, division of labor, and perceived fairness—are inconsistent with what resource theory alone would predict (e.g., Chen, 2018; Hu, 2018; Li et al., 2020; Zhang, 2015; Zuo & Bian, 2001).

Taken together, it is not unusual for wives in modern China to experience discrepancies between relative economic resources and intra-family decision power. Applying our framework to this context, we raise the second hypothesis:

- *Hypothesis 2: (H2): There is an interaction effect of economic dependence and intra-household decision power on married women’s gender ideology, such that among high-independence wives, lower decision power is associated with less egalitarian ideology relative to aligned peers, and among high-dependence wives, higher decision power is associated with more traditional ideology relative to aligned peers.*

## Data and Methods

### Data

This study utilized data from the China Family Panel Studies (CFPS), focusing specifically on the 2014 wave, as it is the only wave that includes the key variable of interest: gender ideology. We will also need data on economic dependence and family power derived from CFPS 2012, 2016, and 2018 for implementing our analytic strategies and/or for supplementary analyses, as the subsequent sections will elaborate on.

The selection of respondents adhered to several criteria. First, we concentrated exclusively on heterosexual and dual-earning couples who are “active labor participants,” restricting the sample to (a) married women aged between 21 and 60 and (b) those reporting an employed status. Next, we further narrowed the sample to married women who had the requisite information about their husbands.

The original sample size of CFPS 2014 is 37,147. We constrain the sample to married women who are aged between 21 and 60 and employed, which results in a sample size of 8,003. We then further limit the sample to those who have required information about their husbands in CFPS and exclude couples where both sides report no income. We then obtain a sample size of 3,351. Among them, 51 people have missing values in family decision-making power, and thus, we end up with a sample of 3,300 in the 2014 cross-sectional data.

Among the 3,300 respondents, 1,648, 1506, and 714 have the income data about both sides of couples in CFPS 2012, CFPS 2016, and CFPS 2018, respectively. Since our analytic strategies require different income data for different hypotheses, the observation number varies accordingly.

### Measures

#### Economic Dependence

Following mainstream practices (e.g., Brines, 1994; Greenstein, 2000), we measure a wife’s economic dependence on her husband using Eq. 1. A higher score means that a married woman is more economically dependent on her husband.

1 $$\:\mathrm{E}\mathrm{c}\mathrm{o}\mathrm{n}\mathrm{o}\mathrm{m}\mathrm{i}\mathrm{c}\:\mathrm{D}\mathrm{e}\mathrm{p}\mathrm{e}\mathrm{n}\mathrm{d}\mathrm{e}\mathrm{n}\mathrm{c}\mathrm{e}=\:\frac{Spous{e}^{{\prime\:}}s\:Income-\:Self\:Income}{Self\:Income\:+\:Spous{e}^{{\prime\:}}s\:Income}$$

#### Gender Ideology

In the recent decade, an important development within family sociology and gender literature is the increasingly recognized multi-dimensional gender ideologies (e.g., Begall et al., 2023; Cotter et al., 2011; Grunow et al., 2018; Knight & Brinton, 2017; van Damme & Pavlopoulos, 2022). Instead of considering traditional ideology and egalitarian ideology as the two ends of a continuous spectrum, research has shown that people can simultaneously hold traditional and egalitarian attitudes in different aspects of paid market labor participation, unpaid housework participation, childrearing, and so forth, and simultaneously hold traditional and egalitarian attitudes for the roles of mother and father, respectively (Grunow et al., 2018; Knight & Brinton, 2017). While the literature on multi-dimensional gender ideologies is still growing, the general pattern is to treat people’s attitudes towards the following aspects: (a) women’s role in the labor market, (b) women’s role in housework, (c) meanings of marriage and children for women, (d) men’s role in the labor market, (e) men’s role in housework, and (f) meanings of marriage and children for men.

There are four items related to gender ideologies in CFPS 2014: (a) Men should focus on career, while women should focus on family; (b) Marrying well is more important for women than doing well; (c) Women should have at least one child; (d) Men should do half of the housework. All items have five Likert scales, from 1= extremely disagree to 5 = extremely agree.

We first reverse-coded the “men housework” item and then examined whether the four items could be combined into a reliable scale of gender ideology. Cronbach’s alpha for the four-item scale was 0.37 (95% CI [0.31, 0.39]), which falls below the commonly accepted threshold of 0.70 for adequate internal consistency. 1 Given this low reliability, we proceeded with the (a) “men career & women family” item only in our main analysis, and a higher score indicates a more traditional gender ideology. We chose such an item because it aligns well with the specific concept of male breadwinning and female homemaking. Meanwhile, we conducted additional analyses by adding all four items into regression models, with results available in Appendices (Table A1). The additional analysis confirms that the other three items are not statistically associated with economic dependence.

#### Household Decision Power

We assessed household decision power using questions about who is responsible for four types of decisions within the household: (1) family expenditure, (2) savings, investment, and insurance, (3) house purchasing, and (4) high-priced product purchasing. If a wife is responsible for any of these decisions, the variable is coded as *one* (46%); otherwise, it is coded as *zero* (54%).

#### Other Variables

Additional covariates included in our models, which will be elaborated in the next section, are education (measured in years of schooling), educational assortative mating types (hypergamy, homogamy, and hypogamy), age, the age difference between couples, number of children, residential location (urban vs. rural), Chinese Communist Party (CCP) membership, and occupation status. Occupation status is measured using the first digit of the Chinese national occupational classification system and is treated as a continuous variable. These covariates were included due to their potential association with women’s economic dependence and gender ideology. Table 1 presents the summary statistics of all variables.

**Table 1:** Summary statistics of variables

Variables	Total *N*	Mean/*N*	SD/%	Range
Economic dependence 2014	3,300	0.30	0.75	−1–1
Traditional gender ideology	3,300	3.86	1.25	1–5
Decision power (original scale)	3,300	1.27	1.62	0–4
Decision power (binary)	3,300			
No		1,792.00	54.30%	
Yes		1,508.00	45.70%	
Occupation status	3,300	4.57	1.41	1–8
Annual Income (RMB)	3,300	13,609.70	21,360.47	0–408,400
Education (years)	3,300	7.90	4.88	0–22
Educational assortative mating	3,300			
Homogamy		1,183	35.85%	
Hypergamy		1,377	41.73%	
Hypogamy		740	22.42%	
Age	3,300	41.96	10.15	21–60
Age difference	3,300	1.89	3.26	−28–39
Children number	3,300	1.41	0.79	0–6
Urban	3,300			
No		1,570	47.58%	
Yes		1,730	52.42%	
Party membership	3,300			
Non-CCP		3,149	95.42%	
CCP		151	4.58%	
Economic dependence 2012	1648	0.32	0.69	−1–1
Economic dependence 2016	1506	0.25	0.45	−1–1
Economic dependence 2018	714	0.19	0.36	−1–1

### Analytic Strategies

Hypothesis 1 predicts a positive association between married women’s traditional gender ideology and economic dependence. While causality is not the primary focus of this study, because we do acknowledge that economic outcomes and ideology will reinforce each other, we aim to provide evidence that the association is not solely driven by the “economic dependence—ideology” direction. For this purpose, we employ a lagged dependent variable (LDV) model to capture the autoregressive process and dynamic relationship between gender ideology and economic dependence.

2 $$\:{ED14}_{i}=a+{b}_{1}{Ideo}_{i}+{b}_{2}{ED12}_{i}+\boldsymbol{b}{\boldsymbol{C}\boldsymbol{o}\boldsymbol{v}1}_{\boldsymbol{i}}+{e}_{i}$$

Equation 2 illustrates our strategy of examining H1, where ED14 denotes economic dependence in CFPS 2014, Ideo denotes traditional gender ideology, ED12 denotes economic dependence in CFPS 2012, and Cov1 indicates other covariates. Following and adapting from prior works (e.g., Bianchi et al., 1999; Sørensen & McLanahan, 1987; Van Berkel & De Graaf, 1998), we include into the covariates both women’s socioeconomic and demographic variables (education, age, and occupational status, party membership, residential type, and number of children) and the difference between couples (educational assortative mating and age gap between couples). One key difference between our study and prior literature is that we do not include the levels of women’s and their husbands’ labor force supply, namely, couples’ absolute and relative worktime, in the covariates in the main analysis. This is because in our conceptualization, women’s and their husbands’ labor participation can be one mechanism underlying gender ideology and economic dependence. However, we conducted an additional analysis (results available in Table A1 of the Appendices), which shows consistent conclusions with the main analysis. We also conducted a Variance Inflation Factor (VIF) diagnostics for all the independent variables included in Eq. 1, and the results show that multicollinearity is not an issue (results available in Appendix B).

3 $$\:{Ideo}_{i}=a+{b}_{1}{ED14}_{i}+{b}_{2}{DP}_{i}+{b}_{3}{DP}_{i}\times\:{ED14}_{i}+\boldsymbol{b}{\boldsymbol{C}\boldsymbol{o}\boldsymbol{v}2}_{\boldsymbol{i}}+{e}_{i}$$

Hypothesis 2 predicts an interaction effect between economic dependence and family decision-making power on married women’s gender ideology. Equation 3 illustrates our strategies for examining H2. DP denotes decision-making power within the family, measured as a binary variable (0 = No, 1 = Yes). Cov2 differs from Cov1 in the sense that Cov2 does not include the couples’ demographic and socioeconomic differences (educational assortative mating and age gap between couples). We make this decision to avoid the potential multicollinearity between economic dependence and couples’ demographic and socioeconomic differences. We also conducted a Variance Inflation Factor (VIF) diagnostics for all the independent variables included in Eq. 3, and the results show that multicollinearity is not an issue (results available in Appendix B). Additional analysis further shows that the conclusions are consistent regardless of whether we include couples’ demographic and socioeconomic differences (results available in Appendix C).

4 $$\:{Ideo}_{i}=a+{b}_{1}{ED14\_bin}_{i}+{b}_{2}{DP}_{i}+{b}_{3}{DP}_{i}\times\:{ED14\_bin}_{i}+\boldsymbol{b}{\boldsymbol{C}\boldsymbol{o}\boldsymbol{v}2}_{\boldsymbol{i}}+{e}_{i}$$

To match our two-by-two conceptual framework, apart from the continuous variable, we also recode economic dependence into a binary variable with two values—high dependence (55%) and low dependence (45%)—using the mean level of dependence (*M* = 0.30) as the reference point. We chose the average level of dependence rather than zero dependence as the reference point to produce more practical implications: Since economically independent wives (i.e., those below zero dependence) are a minority group, further dividing this group by decision power would constrain the practical implications of the analysis. We also conduct a Variance Inflation Factor (VIF) diagnostics for all the independent variables included in Eq. 4, and the results show that multicollinearity is not an issue (results available in Appendix B).

## Results

### Main Analysis

Hypothesis 1 predicts a positive association between married women’s traditional gender ideology and economic dependence. The linear regression results presented in Table 2 consistently support Hypothesis 1. To facilitate the interpretability of coefficients, we have standardized both economic dependence and gender ideology. Model 1 shows that traditional gender ideology is positively associated with economic dependence (*B* = 0.102, *p* < 0.001). Model 2 includes a lagged dependent variable (LDV) and shows consistent results: Traditional gender ideology is positively associated with economic dependence (*B* = 0.084, *p* < 0.001). Taken together, Hypothesis 1 is supported.

**Table 2:** OLS regressions predicting economic dependence 2014

Variable	Model 1	Model 2
Gender ideology	0.102^***^	0.084^***^
	(0.025)	(0.024)
Economic dependence 2012		0.510^***^
		(0.033)
Education (no. years)	−0.031^***^	−0.021^**^
	(0.007)	(0.007)
Educational assortative		
Reference: Homogamy		
Hypergamy	0.101^+^	0.076
	(0.059)	(0.055)
Hypogamy	0.008	−0.007
	(0.064)	(0.060)
Age	0.004	0.005^+^
	(0.003)	(0.003)
Age difference	−0.005	0.001
	(0.008)	(0.007)
No. children	0.023	0.005
	(0.033)	(0.031)
Urban (1 = Yes)	−0.317^***^	−0.238^***^
	(0.052)	(0.048)
Party (1 = CCP)	−0.196^+^	−0.115
	(0.110)	(0.103)
Occupation status	0.025	0.035^+^
	(0.019)	(0.018)
Constant	0.067	−0.258
	(0.187)	(0.176)
Observations	1,648	1,648
R^2^	0.116	0.231
Adjusted R^2^	0.111	0.226
Residual Std. Error	0.943	0.880
F Statistic	21.477^***^	44.726^***^

*Note*: a. + *p*<0.1; * *p*<0.05; ** *p*<0.01; *** p<0.001. Two-tailed testsb. Standard errors in parentheses. ED = Economic dependencec. Economic Dependence and Gender Ideology are standardized

Hypothesis 2 predicts an interaction effect between economic dependence and family decision power on married women’s gender ideology, suggesting that among high-independence wives, lower decision power is associated with less egalitarian ideology relative to aligned peers, and among high-dependence wives, higher decision power is associated with more traditional ideology relative to aligned peers. The results support H2. Models 1 and 2 in Table 3 show results using economic dependence as a continuous variable. While economic dependence is positively associated with traditional gender ideology on average (*B* = 0.04, *p* < 0.05), this finding is expected, given the previously confirmed association in Hypothesis 1. Interestingly, decision power is not significantly associated with gender ideology after controlling for a series of demographic variables (*B* = − 0.020, *p* > 0.1). Two possible explanations are (a) family decision power is not strongly determined by the wife’s gender ideology, and (b) family decision power alone does not shape the wife’s gender ideology.

**Table 3:** OLS regressions predicting traditional gender ideology

Variable	Model 1	Model 2	Model 3	Model 4
ED 14_continous	0.040^*^	0.010		
	(0.017)	(0.022)		
ED 14_binary (1 = Yes)			0.114^***^	0.038
			(0.034)	(0.045)
Decision power (1 = Yes)	−0.020	−0.021	−0.021	−0.117^*^
	(0.033)	(0.033)	(0.033)	(0.049)
ED 14_continous * Decision power (1 = Yes)		0.067^*^		
		(0.032)		
ED 14_binary (1 = Yes) * Decision power (1 = Yes)				0.170^**^
				(0.065)
Education (no. years)	−0.041^***^	−0.042^***^	−0.040^***^	−0.041^***^
	(0.004)	(0.004)	(0.004)	(0.004)
Age	0.016^***^	0.016^***^	0.016^***^	0.016^***^
	(0.002)	(0.002)	(0.002)	(0.002)
No. children	0.101^***^	0.100^***^	0.100^***^	0.098^***^
	(0.021)	(0.021)	(0.021)	(0.021)
Urban (1 = Yes)	−0.093^**^	−0.089^*^	−0.088^*^	−0.082^*^
	(0.036)	(0.036)	(0.036)	(0.036)
Party (1 = CCP)	0.132	0.136^+^	0.134^+^	0.141^+^
	(0.081)	(0.081)	(0.081)	(0.081)
Occupation status	0.053^***^	0.052^***^	0.053^***^	0.052^***^
	(0.013)	(0.013)	(0.013)	(0.013)
Constant	−0.673^***^	−0.675^***^	−0.740^***^	−0.698^***^
	(0.122)	(0.122)	(0.124)	(0.125)
Observations	3,300	3,300	3,300	3,300
R^2^	0.155	0.156	0.156	0.158
Adjusted R^2^	0.153	0.154	0.154	0.156
Residual Std. Error	0.920	0.920	0.920	0.919
F Statistic	75.334^***^	67.501^***^	76.135^***^	68.559^***^

*Note*: a. + *p*<0.1; * *p*<0.05; ** *p*<0.01; *** *p*<0.001. Two-tailed testsb. Standard errors in parentheses. ED = Economic dependencec. Economic Dependence and Gender Ideology are standardized

**Fig. 2:**
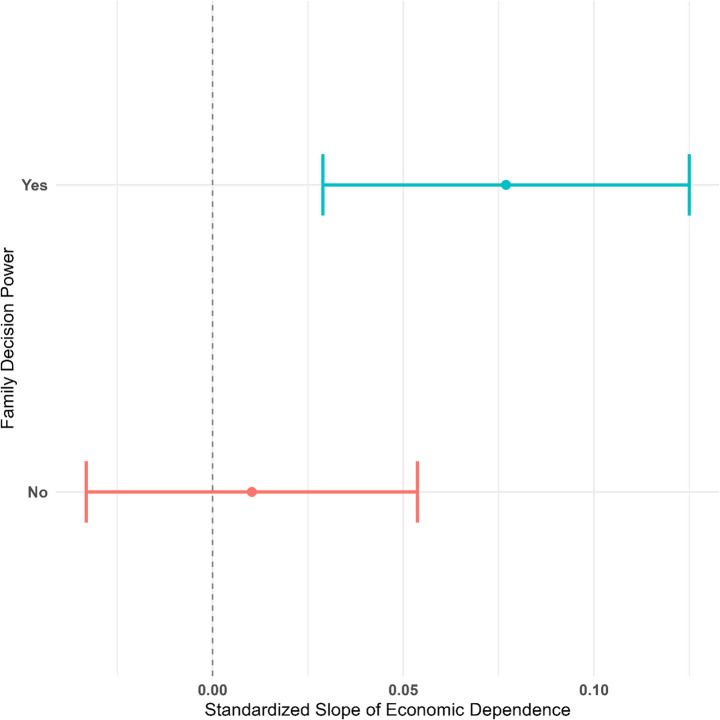
Standardized Coefficients of Economic Dependence on Gender Ideology by Decision Power *Note*: Covariates include education, age, occupational status, party membership, residential type, and number of children

Model 2 shows that there is a significant interaction effect between economic dependence and decision power, such that the association is larger for those with decision power (*B* = 0.067, *p* < 0.05) than those without decision power (*B* = 0.010, *p* > 0.1). Figure 2 illustrates the differences in the association.

**Fig. 3:**
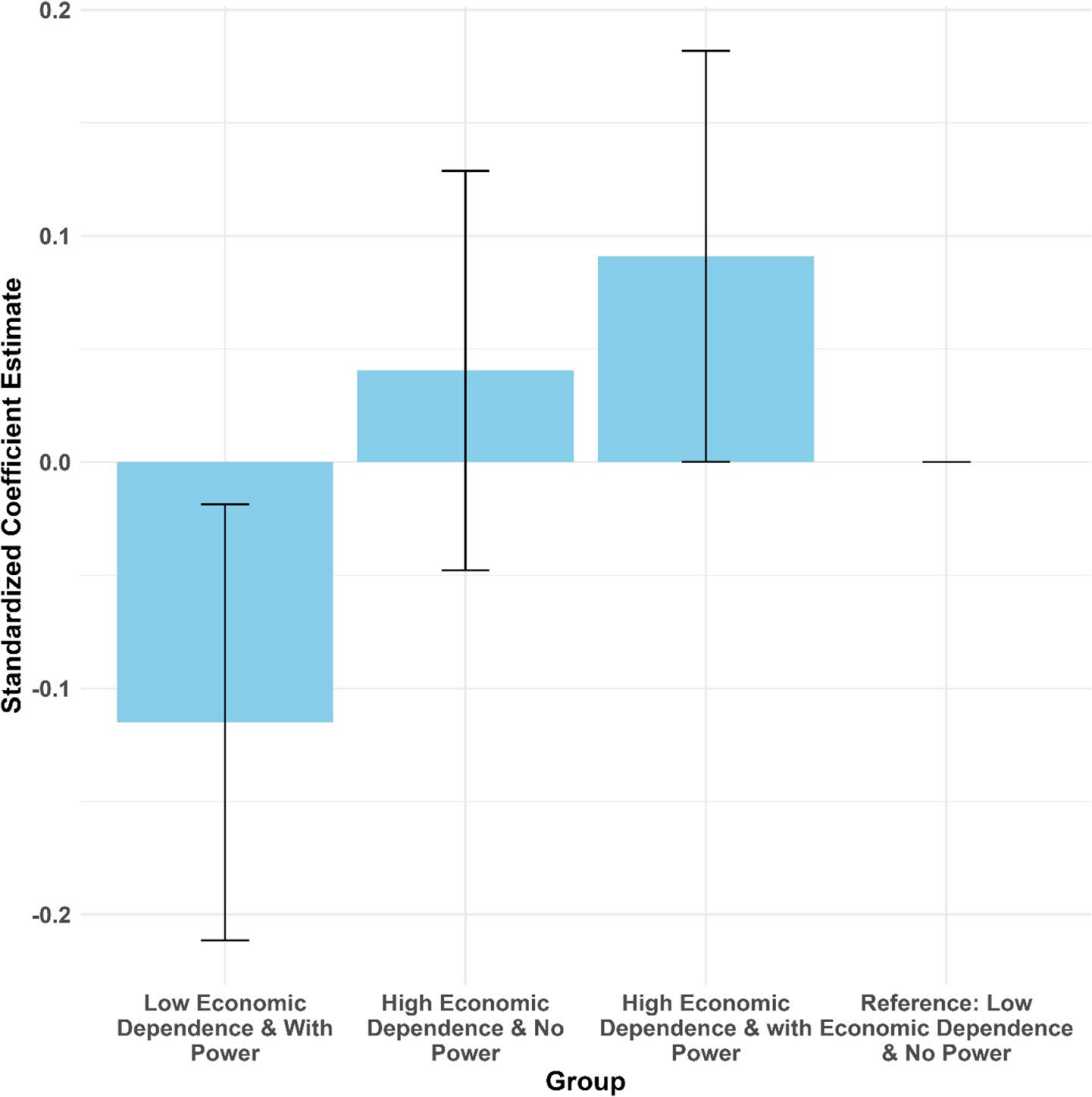
Standardized Coefficients for Interaction between Economic Dependence and Decision Power *Note*: Covariates include education, age, occupational status, party membership, residential type, and number of children

**Fig. 4:**
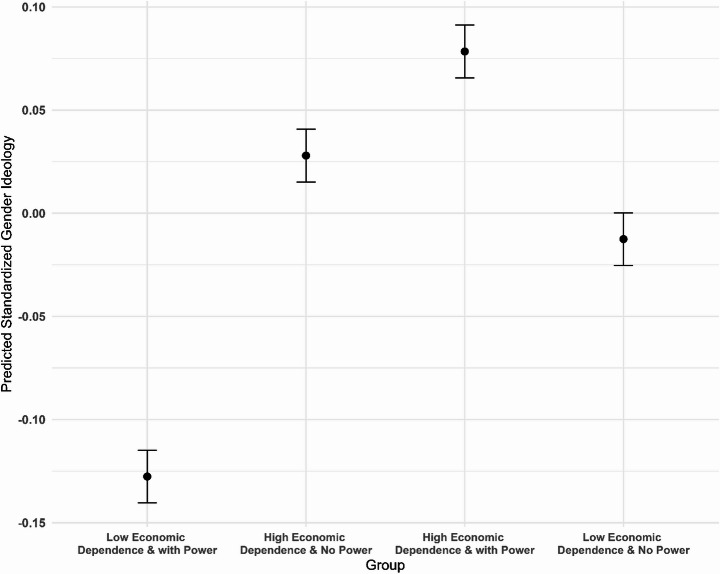
Average Marginal Predictions of Standardized Gender Ideology *Note*: Covariates include education, age, occupational status, party membership, residential type, and number of children

Models 3 and 4 in Table 3 show the regression results when economic dependence is treated as a binary variable (high dependence and low dependence), and Fig. 3 illustrates the differences in association between different groups. To better interpret these results, we also plotted the average marginal predictions of gender ideology, as Fig. 4 shows. From these results, we can see that wives with low economic dependence and high decision power report the lowest scores of traditional gender ideology, while wives with high economic dependence and high decision power report the highest scores, with the other two groups falling in between. This pattern aligns with our expectations. Compared to their counterparts with low economic dependence and high decision power, the lack of decision power represents a penalty for wives with low economic dependence, leading them to show less support for egalitarian ideologies. Similarly, possessing decision power acts as a bonus for wives with high economic dependence, making them more likely to accept the current division of labor and support traditional gender ideologies. Taken together, Hypothesis 2 is supported.

### Robustness Check

To examine the robustness of these findings, we conducted some robustness analysis for both hypotheses. For H1, we replace the original outcome measure of Economic Dependence 2014 with Economic Dependence 2016 and 2018, respectively. Models 1 and 2 in Table 4 show the results. To construct the dataset, we require respondents to appear in all of the 2012, 2014, and 2016 Waves in Model 1 (*N* = 880) and appear in all of the 2012, 2014, and 2018 Waves in Model 2 (*N* = 429).^2^ The results of both models show a consistent positive and significant association between gender ideology and economic dependence (*B* = 0.118, *p* < 0.001; *B* = 0.140, *p* < 0.001) .

**Table 4:** OLS regressions predicting economic dependence 2016 & 2018

Variable	ED 2016	ED 2018
	Model 1	Model 2
Gender ideology	0.118^***^	0.140^**^
	(0.035)	(0.048)
Economic dependence 2012	0.298^***^	0.246^**^
	(0.049)	(0.077)
Education (no. years)	−0.007	0.005
	(0.010)	(0.017)
Educational assortative		
Reference: Homogamy		
Hypergamy	−0.017	−0.007
	(0.082)	(0.116)
Hypogamy	−0.045	−0.313^**^
	(0.087)	(0.119)
Age	0.002	0.010
	(0.004)	(0.007)
Age difference	−0.031^**^	0.015
	(0.011)	(0.019)
No. children	0.060	−0.025
	(0.046)	(0.077)
Urban (1 = Yes)	−0.154^*^	−0.361^***^
	(0.070)	(0.108)
Party (1 = CCP)	−0.044	−0.059
	(0.151)	(0.185)
Occupation status	−0.028	−0.012
	(0.026)	(0.037)
Constant	0.052	−0.052
	(0.270)	(0.413)
Observations	880	429
R^2^	0.124	0.129
Adjusted R^2^	0.113	0.106
Residual Std. Error	0.942	0.946
F Statistic	11.168^***^	5.604^***^

*Note*: a. + *p*<0.1; * *p*<0.05; ** *p*<0.01; *** *p*<0.001. Two-tailed testsb. Standard errors in parentheses. ED = Economic dependencec. Economic Dependence and Gender Ideology are standardized

For H2, we replaced the economic dependence and decision power variables with lagged variables measured in 2012. However, the CFPS 2012 survey does not ask about family decisions in specific aspects, such as (a) family expenditure, (b) savings, investment, and insurance, (c) house purchasing, and (d) high-priced product purchasing. Instead, CFPS 2012 includes a general question, “Who is in charge of important family decisions?” We assigned a value of *one* to decision power if the wife is in charge of important family decisions (19%) and *zero* otherwise (81%). Table 5 presents the regression results. Consistent with the previous results, the association between economic dependence and traditional ideology is stronger for wives with high decision power than for those with low decision power.

**Table 5:** OLS regression predicting traditional gender Ideology, lagged predictors

Variable	Model 1	Model 2	Model 3	Model 4
ED 12_continous	0.055^**^	0.043^*^		
	(0.019)	(0.021)		
ED 12_binary (1 = Yes)			0.164^***^	0.116^**^
			(0.040)	(0.044)
Decision power 12 (1 = Yes)	−0.020	−0.021	−0.021	−0.115^*^
	(0.033)	(0.033)	(0.033)	(0.049)
ED 12_continous * Decision power 12 (1 = Yes)		0.069		
		(0.049)		
ED 12_binary (1 = Yes) * Decision power 12 (1 = Yes)				0.255^**^
				(0.096)
Education (no. years)	−0.035^***^	−0.035^***^	−0.034^***^	−0.034^***^
	(0.005)	(0.005)	(0.005)	(0.005)
Age	0.021^***^	0.022^***^	0.021^***^	0.021^***^
	(0.002)	(0.002)	(0.002)	(0.002)
No. children	0.108^***^	0.108^***^	0.106^***^	0.106^***^
	(0.025)	(0.025)	(0.025)	(0.025)
Urban (1 = Yes)	−0.126^**^	−0.125^**^	−0.116^**^	−0.115^**^
	(0.042)	(0.042)	(0.042)	(0.042)
Party (1 = CCP)	0.150	0.152	0.160	0.161
	(0.098)	(0.098)	(0.098)	(0.098)
Occupation status	−0.050^**^	−0.049^**^	−0.050^**^	−0.047^**^
	(0.017)	(0.017)	(0.017)	(0.017)
Constant	−0.554^***^	−0.564^***^	−0.652^***^	−0.648^***^
	(0.144)	(0.144)	(0.146)	(0.146)
Observations	2,439	2,439	2,439	2,439
R^2^	0.149	0.149	0.152	0.154
Adjusted R^2^	0.146	0.146	0.149	0.151
Residual Std. Error	0.924	0.924	0.923	0.921
F Statistic	53.058^***^	47.401^***^	54.349^***^	49.207^***^

*Note*: a. + *p*<0.1; * *p*<0.05; ** *p*<0.01; *** *p*<0.001. Two-tailed testsb. Standard errors in parentheses. ED = Economic dependencec. Economic Dependence and Gender Ideology are standardizedd. The attrition between the 2012 and 2014 waves and the missing values in ED 12 andDecision Power 12 result in a sample reduction from 3300 to 2439

**Fig. 5:**
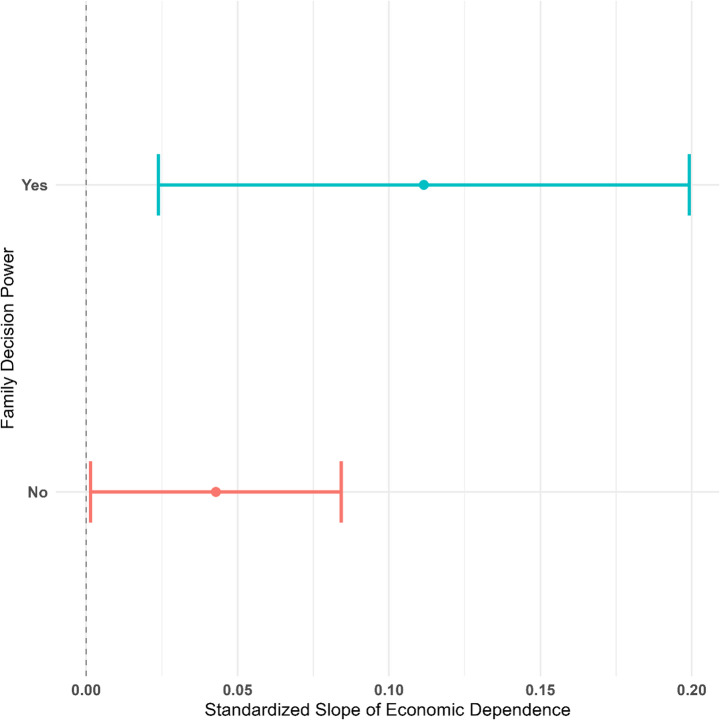
Standardized Coefficients of Economic Dependence on Gender Ideology by Decision Power, Lagged Predictors *Note*: Covariates include education, age, occupational status, party membership, residential type, and number of children

However, it is noticeable that the interactive effects are smaller than those in the main analysis. Take the results of continuous Economic dependence as an example. The slope for wives with low decision power changes from non-significance to significance (*B* = 0.043, *p* < 0.05), although it is much smaller than the slope for wives with high decision power (*B* = 0.112). Figure 5 illustrates the pattern. The smaller gaps between with-power and no-power groups are likely due to different coding practices: Because of the broader question on decision-making power in 2012, much fewer wives (less than 20%) are classified as “with power,” compared to nearly half in CFPS 2014. As a result, some wives categorized as “with power” in CFPS 2014 may have been categorized as “without power” in CFPS 2012, thereby reducing the gaps between the groups.

Despite this, the results derived from the 2012 lagged variables provide valuable supplementary evidence that the interaction between economic resources and family decision power shapes married women’s gender ideology. 

## Discussions and Conclusions

Our results suggest a key mechanism through which two important inequality indicators, gender ideology and economic dependence, reinforce each other within marriage: married women with traditional gender ideologies are more likely to be economically dependent on their husbands, and this economic dependence interacts with decision-making power within the family to shape gender ideology.

In the context of modern China, the past half-century has witnessed a regression in gender equality in the labor market, alongside a resurgence of Confucian patriarchal traditions that confine women to caregiving and supportive roles within the family (Ji et al., 2017). Confucian patriarchal values encourage people to divide labor along traditional lines, whether through internalized beliefs or to avoid social sanctions. Our study provides empirical evidence supporting this relationship regarding internalized beliefs.

Furthermore, people’s daily experiences and the benefits they derive from their life arrangements can reinforce or alter their gender ideologies (Bolzendahl & Myers, 2004; Davis & Robinson, 1991). In China, it is not uncommon for wives to experience discrepancies between their relative economic resources and intra-family decision-making power. Although wives’ disproportionate domestic work is inherently associated with economic dependence, it can also help them gain decision-making power within the family (e.g., Pimentel, 2006; Sevilla et al., 2012; Zuo & Bian, 2005). Meanwhile, Confucian patriarchal values suggest that even economically independent wives may not necessarily enjoy corresponding decision-making power within the family. By examining the discrepancy between economic resources and family decision-making power, our study provides novel empirical evidence on how the labor and domestic spheres can jointly shape married women’s gender ideologies.

Taken together, our findings emphasize individual agency in the interplay between gender ideologies and gender disparities. While economic disparities and dependence within couples may be influenced by external and institutional factors, agency manifests in the fact that family power distribution need not align with economic resource distribution and that women’s acceptance of gender roles can change in accordance with their perceived benefits.

As is customary, this study has certain limitations. First, we do not claim causality for two reasons. Conceptually, we acknowledge the mutual influence between economic resources and ideologies. Additionally, the limitations of cross-sectional data restrict causal claims. Although the CFPS is longitudinal, only the 2014 wave includes questions regarding gender ideologies. While we have made efforts to clarify the direction of influences by leveraging temporal order and employing lagged variables, we recognize the limited ability to disentangle the directions of influence between economic resources and ideologies. If future CFPS waves include measures of gender ideologies, further studies could investigate these mutual influences in greater depth.

Second, we acknowledge the boundary conditions related to time and regions and call attention to the generalizability of the results. For the former, we notice that the average economic dependence of married women keeps declining (from 0.32 in the 2012 Wave to 0.19 in the 2018 Wave), based on our employed sample. Under this context, it is particularly rewarding for future studies to investigate who the remaining group with high dependence is and explore whether the high dependence is mainly due to their ideology, structural inequalities, or both. For the latter, it will be interesting to conduct a multinational comparison that includes different cultures and regimes.

Third, among the four gender ideology items available in CFPS 2014, we selected the statement *“Men should focus on career*,* while women should focus on family”* as our measure of traditional gender ideology. This decision was guided by two considerations. First, from a multidimensional perspective, this item most directly reflects our substantive focus on the breadwinner–homemaker divide. Second, internal consistency tests indicated that relying on this single item was the most appropriate strategy. We acknowledge, however, that this approach necessarily constrains the scope of construct validation.

Finally, this study examines a particular type of personal interest and experience: the discrepancy or alignment between personal economic resources and family decision-making power. Future studies could focus on how other personal interests and experiences shape gender ideologies. For instance, exploring the discrepancy or alignment between family decision-making power and other socioeconomic resources, such as personal educational attainment or a couple’s parental wealth, could provide interesting insights.

## Figures and Tables

**Fig. 6:**
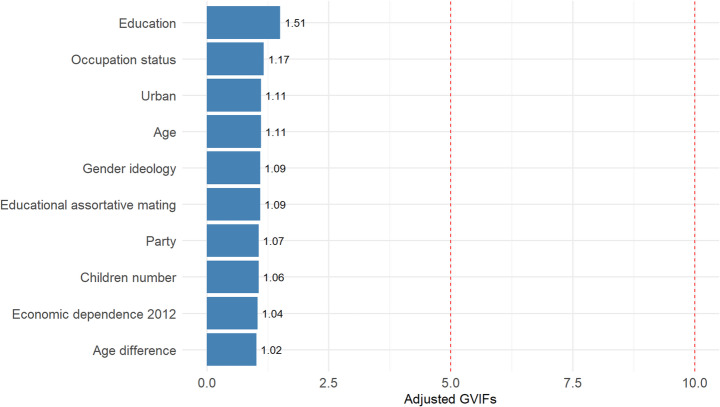
Variance Inflation Factor Diagnostic Results for Eq. 1 Note: We assessed multicollinearity using variance inflation factors (VIFs), reporting the adjusted generalized VIF ($\:{\mathrm{G}\mathrm{V}\mathrm{I}\mathrm{F}}_{\mathrm{a}\mathrm{d}\mathrm{j}}={\mathrm{G}\mathrm{V}\mathrm{I}\mathrm{F}}^{1/\left(2\times\:\mathrm{D}\mathrm{f}\right)}$) to allow comparability across predictors with differing degrees of freedom

**Fig. 7:**
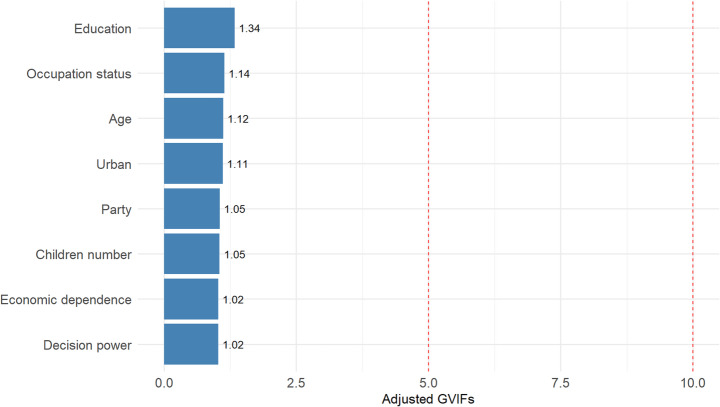
Variance Inflation Factor Diagnostic Results for Eq. 2 Note: We assessed multicollinearity using variance inflation factors (VIFs), reporting the adjusted generalized VIF ($\:{\mathrm{G}\mathrm{V}\mathrm{I}\mathrm{F}}_{\mathrm{a}\mathrm{d}\mathrm{j}}={\mathrm{G}\mathrm{V}\mathrm{I}\mathrm{F}}^{1/\left(2\times\:\mathrm{D}\mathrm{f}\right)}$) to allow comparability across predictors with differing degrees of freedom

**Fig. 8:**
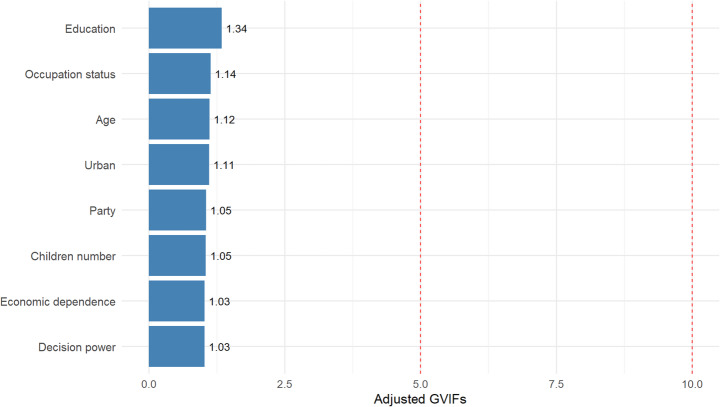
Variance Inflation Factor Diagnostic Results for Eq. 3 Note: We assessed multicollinearity using variance inflation factors (VIFs), reporting the adjusted generalized VIF ($\:{\mathrm{G}\mathrm{V}\mathrm{I}\mathrm{F}}_{\mathrm{a}\mathrm{d}\mathrm{j}}={\mathrm{G}\mathrm{V}\mathrm{I}\mathrm{F}}^{1/\left(2\times\:\mathrm{D}\mathrm{f}\right)}$) to allow comparability across predictors with differing degrees of freedom

## Data Availability

The data underlying this article are from the China Family Panel Studies, which are conducted by the Institute of Social Science Survey, Peking University, China. The authors are solely responsible for the analyses and interpretations of the data. The data were provided by the Institute of Social Science Survey, Peking University, China, by permission, and data will be shared on request to the corresponding author with the permission of the Institute of Social Science Survey, Peking University, China.

## Appendix 1. Additional Analysis for Hypothesis 1

**Table 6:** OLS regression predicting economic dependence

Variable	ED 2014		ED 2016	ED 2018
	(1)	(2)	(3)	(4)
Gender ideologies				
Men career & women family	0.082^**^	0.070^**^	0.124^**^	0.171^**^
	(0.026)	(0.025)	(0.039)	(0.057)
Marriage importance	0.006	0.006	−0.022	−0.048
	(0.020)	(0.019)	(0.030)	(0.045)
Child importance	−0.007	−0.004	−0.016	−0.017
	(0.023)	(0.021)	(0.035)	(0.049)
Men housework	0.008	0.005	−0.027	0.040
	(0.022)	(0.021)	(0.035)	(0.048)
Economic dependence 2012		0.424^***^	0.246^***^	0.219^**^
		(0.031)	(0.051)	(0.080)
Work hours (weekly)	−0.010^***^	−0.009^***^	−0.004^**^	−0.005^*^
	(0.001)	(0.001)	(0.001)	(0.002)
Work hours of husbands (weekly)	0.012^***^	0.011^***^	0.004^**^	−0.002
	(0.001)	(0.001)	(0.001)	(0.002)
Education (no. years)	−0.022^**^	−0.015^*^	−0.010	0.007
	(0.007)	(0.007)	(0.011)	(0.018)
Educational assortative				
Reference: Homogamy				
Hypergamy	0.099^+^	0.076	−0.010	−0.022
	(0.054)	(0.051)	(0.083)	(0.120)
Hypogamy	−0.014	−0.027	−0.033	−0.345^**^
	(0.059)	(0.056)	(0.088)	(0.122)
Age	0.007^*^	0.007^**^	0.003	0.011
	(0.003)	(0.003)	(0.004)	(0.007)
Age difference	0.006	0.009	−0.028^*^	0.021
	(0.007)	(0.007)	(0.011)	(0.020)
No. children	0.013	0.001	0.044	−0.046
	(0.033)	(0.031)	(0.051)	(0.087)
Urban (1 = Yes)	−0.179^***^	−0.130^**^	−0.129^+^	−0.331^**^
	(0.049)	(0.047)	(0.073)	(0.113)
Party (1 = CCP)	−0.259^*^	−0.178^+^	−0.051	−0.151
	(0.102)	(0.097)	(0.152)	(0.188)
Occupation status	0.022	0.032^+^	−0.030	−0.018
	(0.018)	(0.017)	(0.027)	(0.038)
Province fixed effect	Yes	Yes	Yes	Yes
Constant	−0.763^*^	−0.799^*^	0.353	0.177
	(0.358)	(0.339)	(0.551)	(0.719)
Observations	1,648	1,648	880	429
R^2^	0.276	0.351	0.163	0.202
Adjusted R^2^	0.258	0.335	0.123	0.119
Residual Std. Error	0.861	0.816	0.937	0.938
F Statistic	15.351^***^	21.216^***^	4.071^***^	2.452^***^

a. + *p* < 0.1; * *p* < 0.05; ** *p* < 0.01; *** *p* < 0.001. Two-tailed tests

b. Standard errors in parentheses. ED = Economic dependence

c. Economic Dependence and Gender Ideology are standardized

d. Marriage importance: Marrying well is more important for women than doing well; Child importance: Women should have at least one child; Men housework: Men should do half of the housework. All from 1 (extremely disagree) to 5 (extremely agree)

e. To avoid sample reduction caused by the missing values in work hours and enhance the comparability with the main analysis, we impute the missing values with 0. We alternatively tried to use the mean and median as the imputed values, and the coefficients of gender ideologies only slightly changed

## Appendix 3. Additional Analysis for Hypothesis 1 Including Couples’ Differences as Independent Variables

Table 7

**Table 7:** OLS regression predicting traditional gender ideology

Variable	Model 1	Model 2	Model 3	Model 4
ED 14_continous	0.040^*^	0.011		
	(0.017)	(0.022)		
ED 14_binary (1 = Yes)			0.115^***^	0.040
			(0.034)	(0.045)
Decision power (1 = Yes)	−0.020	−0.021	−0.021	−0.115^*^
	(0.033)	(0.033)	(0.033)	(0.049)
ED 14_continous * Decision power (1 = Yes)		0.065^*^		
		(0.032)		
ED 14_binary (1 = Yes) * Decision power (1 = Yes)				0.167^*^
				(0.065)
Education (no. years)	−0.041^***^	−0.041^***^	−0.040^***^	−0.040^***^
	(0.005)	(0.005)	(0.005)	(0.005)
Educational assortative				
Hypergamy	−0.006	−0.007	−0.005	−0.007
	(0.039)	(0.039)	(0.039)	(0.039)
Hypogamy	−0.046	−0.048	−0.047	−0.049
	(0.044)	(0.044)	(0.044)	(0.044)
Age	0.016^***^	0.016^***^	0.016^***^	0.016^***^
	(0.002)	(0.002)	(0.002)	(0.002)
Age difference	−0.007	−0.006	−0.007	−0.006
	(0.005)	(0.005)	(0.005)	(0.005)
No. children	0.100^***^	0.099^***^	0.099^***^	0.097^***^
	(0.021)	(0.021)	(0.021)	(0.021)
Urban (1 = Yes)	−0.096^**^	−0.092^*^	−0.090^*^	−0.085^*^
	(0.036)	(0.036)	(0.036)	(0.036)
Party (1 = CCP)	0.135^+^	0.139^+^	0.138^+^	0.145^+^
	(0.081)	(0.081)	(0.081)	(0.081)
Occupation status	0.053^***^	0.052^***^	0.053^***^	0.052^***^
	(0.013)	(0.013)	(0.013)	(0.013)
Constant	−0.649^***^	−0.651^***^	−0.717^***^	−0.676^***^
	(0.131)	(0.131)	(0.133)	(0.134)
Observations	3,300	3,300	3,300	3,300
R^2^	0.156	0.157	0.157	0.159
Adjusted R^2^	0.153	0.154	0.154	0.156
Residual Std. Error	0.920	0.920	0.920	0.919
F Statistic	55.078^***^	50.870^***^	55.676^***^	51.669^***^

a. + p < 0.1; * p < 0.05; ** p < 0.01; *** p < 0.001. Two-tailed tests

b. Standard errors in parentheses. ED = Economic dependence

b. Standard errors in parentheses. ED = Economic dependence

## Footnotes

1 If we do not reverse-code the “men housework” item, Cronbach’s alpha for the four-item scale was 0.61 (95% CI [0.59, 0.63]), which is still below the commonly accepted threshold of 0.70. More importantly, the different results between the two practices imply that many people have an egalitarian ideology in men’s housework participation but have a traditional ideology in other aspects, which confirms the muti-dimensional perspective.

2 The attrition is high because of the missed values in income in these two waves.

## References

[CR1] Becker, G. S. (1981). *A Treatise on the Family*. Harvard University Press.

[CR2] Begall, K., Grunow, D., & Buchler, S. (2023). Multidimensional gender ideologies across Europe: Evidence from 36 countries. *Gender & Society,**37*(2), 177–207. 10.1177/08912432231155914

[CR3] Bianchi, S. M., Casper, L. M., & Peltola, P. K. (1999). A cross-national look at married women’s earnings dependency. *Gender Issues*, *17*(3), 3–33. 10.1007/s12147-999-0001-0 10.1007/s12147-999-0001-0 12295520

[CR4] Bittman, M., England, P., Sayer, L., Folbre, N., & Matheson, G. (2003). When does gender Trump money? Bargaining and time in household work. *American Journal of Sociology*, *109*(1), 186–214. 10.1086/378341

[CR5] Blau, P. M. (1964). *Exchange and power in social life*. Wiley.

[CR6] Bolzendahl, C. I., & Myers, D. J. (2004). Feminist attitudes and support for gender equality: Opinion change in women and men, 1974–1998*. *Social Forces,**83*(2), 759–789. 10.1353/sof.2005.0005

[CR7] Bourdieu, P. (1984). *Distinction: A social critique of the judgement of taste*. Harvard University Press.

[CR8] Bourdieu, P. (1986). The forms of capital. In J. Richardson (Ed.), *Handbook of Theory and Research for the Sociology of Education* (pp. 241–258). Greenwood.

[CR9] Brayfield, A. A. (1992). Employment resources and housework in Canada. *Journal of Marriage and Family*, *54*(1), 19–30. 10.2307/353272

[CR10] Brines, J. (1994). Economic dependency, gender, and the division of labor at home. *American Journal of Sociology,**100*(3), 652–688. 10.1086/230577

[CR11] Carlsson, F., Martinsson, P., Qin, P., & Sutter, M. (2009). Household decision making and the influence of spouses’ Income, Education, and communist party membership: A field experiment in rural China. *SSRN Scholarly Paper 1395246*. 10.2139/ssrn.1395246

[CR12] Cech, E. A. (2013a). Ideological wage inequalities? The technical/social dualism and the gender wage gap in engineering. *Social Forces,**91*(4), 1147–1182. 10.1093/sf/sot024

[CR13] Cech, E. A. (2013b). The self-expressive edge of occupational sex segregation. *American Journal of Sociology,**119*(3), 747–789. 10.1086/673969

[CR14] Cech, E. A. (2016). Mechanism or myth?: Family plans and the reproduction of occupational gender segregation. *Gender & Society,**30*(2), 265–288. 10.1177/0891243215608798

[CR15] Chen, M. (2018). Does marrying well count more than career? Personal achievement, marriage, and happiness of married women in urban China. *Chinese Sociological Review,**50*(3), 240–274. 10.1080/21620555.2018.1435265

[CR16] Cheng, C., & Xie, Y. (2023). Towards an extended resource theory of marital power: Parental education and household decision-making in rural China. *European Sociological Review,* , Article jcad032. 10.1093/esr/jcad032 10.1093/esr/jcad032 PMC11451950 39371592

[CR17] Chi, W., & Li, B. (2014). Trends in China’s gender employment and pay gap: Estimating gender pay gaps with employment selection. *Journal of Comparative Economics,**42*(3), 708–725. 10.1016/j.jce.2013.06.008

[CR18] Chiappori, P.-A., Iyigun, M., Lafortune, J., & Weiss, Y. (2017). Changing the rules midway: The impact of granting alimony rights on existing and newly formed partnerships. *The Economic Journal,**127*(604), 1874–1905. 10.1111/ecoj.12385

[CR19] Cook, K. S., & Emerson, R. M. (1987). *Social exchange theory*. Sage.

[CR20] Cotter, D., Hermsen, J. M., & Vanneman, R. (2011). The end of the gender revolution? Gender role attitudes from 1977 to 2008. *American Journal of Sociology,**117*(1), 259–289. 10.1086/658853 10.1086/658853 22003521

[CR21] Davis, S. N., & Greenstein, T. N. (2009). Gender ideology: Components, predictors, and consequences. *Annual Review of Sociology,**35*(1), 87–105. 10.1146/annurev-soc-070308-115920

[CR22] Davis, N. J., & Robinson, R. V. (1991). Men’s and women’s consciousness of gender inequality: Austria, West Germany, Great Britain, and the United States. *American Sociological Review,**56*(1), 72–84. 10.2307/2095674

[CR23] Emerson, R. M. (1962). Power-dependence relations. *American Sociological Review,**27*(1), 31–41. 10.2307/2089716

[CR24] Emerson, R. M. (1976). Social exchange theory. *Annual Review of Sociology*, *2*(1), 335–362. 10.1146/annurev.so.02.080176.002003

[CR25] Festinger, L. (1962). *A theory of cognitive dissonance*. Stanford University Press.

[CR26] Folbre, N. (1995). Holding hands at midnight: The paradox of caring labor. *Feminist Economics*, *1*(1), 73–92. 10.1080/714042215

[CR27] Greenstein, T. N. (2000). Economic dependence, gender, and the division of labor in the home: A replication and extension. *Journal of Marriage and Family,**62*(2), 322–335. 10.1111/j.1741-3737.2000.00322.x

[CR28] Grossbard-Shechtman, A. (1984). A theory of allocation of time in markets for labour and marriage. *The Economic Journal*, *94*(376), 863–882. 10.2307/2232300

[CR29] Grunow, D., Begall, K., & Buchler, S. (2018). Gender ideologies in europe: A multidimensional framework. *Journal of Marriage and Family*, *80*(1), 42–60. 10.1111/jomf.12453 29491532 10.1111/jomf.12453 PMC5817238

[CR30] Gupta, S. (2007). Autonomy, dependence, or display? The relationship between married women’s earnings and housework. *Journal of Marriage and Family,**69*(2), 399–417. 10.1111/j.1741-3737.2007.00373.x

[CR31] Gustafsson, B., & Li, S. (2000). Economic transformation and the gender earnings gap in urban China. *Journal of Population Economics*, *13*(2), 305–329. 10.1007/s001480050140

[CR32] He, G., & Wu, X. (2017). Marketization, occupational segregation, and gender earnings inequality in urban China. *Social Science Research*, *65*(C), 96–111. 10.1016/j.ssresearch.2016.12.001 28599783 10.1016/j.ssresearch.2016.12.001

[CR33] He, G., & Wu, X. (2021). Family status and women’s career mobility during urban china’s economic transition. *Demographic Research*, *44*, 189–224. 34054338 10.4054/demres.2021.44.8 PMC8153691

[CR34] Homans, G. C. (1958). Social behavior as exchange. *American Journal of Sociology*, *63*(6), 597–606. 10.1086/222355

[CR35] Horne, C., & Mollborn, S. (2020). Norms: An integrated framework. *Annual Review of Sociology*, *46*(1), 467–487. 10.1146/annurev-soc-121919-054658

[CR36] Hu, Y. (2018). Patriarchal hierarchy? Gender, children’s housework time, and family structure in Post-Reform China. *Chinese Sociological Review,**50*(3), 310–338. 10.1080/21620555.2018.1430508

[CR37] Ji, Y., & Wu, X. (2018). New gender dynamics in Post-Reform china: Family, education, and labor market. *Chinese Sociological Review,**50*(3), 231–239. 10.1080/21620555.2018.1452609

[CR38] Ji, Y., Wu, X., Sun, S., & He, G. (2017). Unequal care, unequal work: Toward a more comprehensive understanding of gender inequality in Post-Reform urban China. *Sex Roles,**77*(11–12), 765–778. 10.1007/s11199-017-0751-1

[CR39] Jost, J. T. (2019). A quarter century of system justification theory: Questions, answers, criticisms, and societal applications. *British Journal of Social Psychology*, *58*(2), 263–314. 10.1111/bjso.12297

[CR40] Jost, J. T., & Banaji, M. R. (1994). The role of stereotyping in system-justification and the production of false consciousness. *British Journal of Social Psychology,**33*(1), 1–27. 10.1111/j.2044-8309.1994.tb01008.x

[CR41] Kan, M. Y., & He, G. (2018). Resource bargaining and gender display in housework and care work in modern China. *Chinese Sociological Review*, *50*(2), 188–230. 10.1080/21620555.2018.1430506

[CR42] Kan, M. Y., & Zhou, M. (2022). Gender and intergenerational support in East Asian families. *Chinese Sociological Review*, *54*(4), 333–341. 10.1080/21620555.2022.2109143

[CR43] Kan, M. Y., Zhou, M., Negraia, D. V., Kolpashnikova, K., Hertog, E., Yoda, S., & Jun, J. (2021). How do older adults spend their time? Gender gaps and educational gradients in time use in East Asian and Western countries. *Journal of Population Ageing*, *14*(4), 537–562. 10.1007/s12062-021-09345-3

[CR44] Kan, M. Y., Zhou, M., Kolpashnikova, K., Hertog, E., Yoda, S., & Jun, J. (2022). Revisiting the gender revolution: Time on paid work, domestic work, and total work in East Asian and Western societies 1985–2016. *Gender & Society*, *36*(3), 368–396. 10.1177/08912432221079664

[CR45] Kanji, S., & Schober, P. (2014). Are couples with young children more likely to split up when the mother is the main or an equal earner? *Sociology*, *48*(1), 38–58. 10.1177/0038038512467710

[CR46] Khoudja, Y., & Fleischmann, F. (2018). Gender ideology and women’s labor market transitions within couples in the Netherlands. *Journal of Marriage and Family*, *80*(5), 1087–1106. 10.1111/jomf.12510

[CR47] Knight, C. R., & Brinton, M. C. (2017). One egalitarianism or several? Two decades of Gender-Role attitude change in Europe. *American Journal of Sociology*, *122*(5), 1485–1532. 10.1086/689814

[CR48] Lennon, M., & Rosenfield, S. (1994). Relative fairness and the division of housework: The importance of options. *American Journal of Sociology,**100*(2), 506–531. 10.1086/230545

[CR49] Li, X., Cao, H., Curran, M. A., Fang, X., & Zhou, N. (2020). Traditional gender ideology, work family conflict, and marital quality among Chinese dual-earner couples: A moderated mediation model. *Sex Roles,**83*(9), 622–635. 10.1007/s11199-020-01125-1

[CR50] Lundberg, S., & Pollak, R. A. (1993). Separate spheres bargaining and the marriage market. *Journal of Political Economy*, *101*(6), 988–1010. 10.1086/261912

[CR51] Lundberg, S., & Pollak, R. A. (1996). Bargaining and distribution in marriage. *Journal of Economic Perspectives*, *10*(4), 139–158. 10.1257/jep.10.4.139

[CR52] Manser, M., & Brown, M. (1980). Marriage and household decision-making: A bargaining analysis. *International Economic Review,**21*(1), 31–44. 10.2307/2526238

[CR53] McElroy, M. B., & Horney, M. J. (1981). Nash-bargained household decisions: Toward a generalization of the theory of demand. *International Economic Review,**22*(2), 333–349. 10.2307/2526280

[CR54] Pimentel, E. E. (2006). Gender ideology, household behavior, and backlash in urban China. *Journal of Family Issues,**27*(3), 341–365. 10.1177/0192513X05283507

[CR55] Qian, Y., & Jin, Y. (2018). Women’s fertility autonomy in urban china: The role of couple dynamics under the universal two-child policy. *Chinese Sociological Review,**50*(3), 275–309. 10.1080/21620555.2018.1428895

[CR56] Rhodebeck, L. A. (1996). The structure of men’s and women’s feminist orientations: Feminist identity and feminist opinion. *Gender & Society,**10*(4), 386–403. 10.1177/089124396010004003

[CR57] Ridgeway, C. L., & Correll, S. J. (2004). Unpacking the gender system: A theoretical perspective on gender beliefs and social relations. *Gender & Society*, *18*(4), 510–531. 10.1177/0891243204265269

[CR58] Ridgeway, C. L., & Smith-Lovin, L. (1999). The gender system and interaction. *Annual Review of Sociology,**25*(1), 191–216. 10.1146/annurev.soc.25.1.191

[CR59] Seron, C., Silbey, S. S., Cech, E. A., & Rubineau, B. (2016). Persistence is cultural: Professional socialization and the reproduction of sex segregation. *Work and Occupations,**43*(2), 178–214. 10.1177/0730888415618728

[CR60] Sevilla, A., Gimenez-Nadal, J. I., & Gershuny, J. (2012). Leisure inequality in the United States: 1965–2003. *Demography,**49*(3), 939–964. 10.1007/s13524-012-0100-5 22589003 10.1007/s13524-012-0100-5

[CR61] Shu, X., Zhu, Y., & Zhang, Z. (2013). Patriarchy, resources, and specialization: Marital decision-making power in urban China. *Journal of Family Issues,**34*(7), 885–917. 10.1177/0192513X12450001

[CR62] Sørensen, A., & McLanahan, S. (1987). Married women’s economic dependency, 1940–1980. *American Journal of Sociology,**93*(3), 659–687. 10.1086/228792

[CR63] Stevenson, B. (2007). The impact of divorce laws on marriage-specific capital. *Journal of Labor Economics,**25*(1), 75–94. 10.1086/508732

[CR64] Stratton, L. S. (2020). The determinants of housework time. *IZA World of Labor*, *133*. 10.15185/izawol.133.v2

[CR65] Van Berkel, M., & De Graaf, N. D. (1998). Married women’s economic dependency in the Netherlands, 1979–1991. *The British Journal of Sociology,**49*(1), 97–117. 10.2307/591265 9569773

[CR66] van Damme, M., & Pavlopoulos, D. (2022). Gender ideology in europe: Plotting normative types in a multidimensional space. *Social Indicators Research*, *164*(2), 861–891. 10.1007/s11205-022-02976-9

[CR67] Wu, Y., & Zhou, D. (2015). Women’s labor force participation in urban China, 1990–2010. *Chinese Sociological Review*, *47*(4), 314–342. 10.1080/21620555.2015.1036234

[CR68] Xu, X., & Lai, S. C. (2002). Resources, gender ideologies, and marital power: The case of Taiwan. *Journal of Family Issues,**23*(2), 209–245. 10.1177/0192513X02023002003

[CR69] Yeung, W. J. J., & Hu, S. (2016). Paradox in marriage values and behavior in contemporary China. *Chinese Journal of Sociology*, *2*(3), 447–476. 10.1177/2057150X16659019

[CR70] Zelditch, M. (2001). Processes of legitimation: Recent developments and new directions. *Social Psychology Quarterly*, *64*(1), 4–17. 10.2307/3090147

[CR71] Zhang, H. (2015). Wives’ relative income and marital quality in urban china: Gender role attitudes as a moderator. *Journal of Comparative Family Studies*, *46*(2), 203–220. 10.3138/jcfs.46.2.203

[CR72] Zhou, M., Kan, M. Y., & He, G. (2022). Intergenerational co-residence and young couple’s time use in China. *Chinese Sociological Review*, *54*(4), 401–431. 10.1080/21620555.2021.1972285

[CR73] Zuo, J., & Bian, Y. (2001). Gendered resources, division of housework, and perceived fairness—A case in urban China. *Journal of Marriage and Family,**63*(4), 1122–1133. 10.1111/j.1741-3737.2001.01122.x

[CR74] Zuo, J., & Bian, Y. (2005). Beyond resources and patriarchy: Marital construction of family decision-making power in Post-Mao urban China. *Journal of Comparative Family Studies,**36*(4), 601–622. 10.3138/jcfs.36.4.601

